# The Goodenough Report on Medical Education

**Published:** 1944

**Authors:** 


### THE GOODENOUGH REPORT ON MEDICAL EDUCATION

*A Summary*

---

THIS Report of the Inter-Departmental Committee on Medical Schools is both interesting and important. Some readers will receive it with surprise at the innovations it suggests, others will think it is not progressive enough. But no Committee can ever issue a report that is really ahead of the general views of the public whose witnesses it has examined. The members of the Committee and its Chairman, Sir William Goodenough, are well known and well trusted both by the public and the medical profession. The most interesting reading comes at the end in Appendix P, where the names of the witnesses are recorded.

Every university in the United Kingdom and all the teaching hospitals submitted evidence, as well as all the licensing Corporations and Royal Colleges, Municipal Corporations, County Councils, the G.M.C., B.M.A., the British Hospitals Association, the Medical Research Council, Medical Students Associations of England and Wales, and Scotland. The Pathological Society, British Pædiatric Association and the 1942 Club were all heard, and in addition thirty-seven independent witnesses. Many other professional organizations and hospitals sent written evidence, but were not heard orally.

From this plethora of witnesses there has emerged a Report signed unanimously by the members of the Committee, without any dissentient or unsatisfied voices. This in itself is remarkable, but it probably results from a marked degree of unanimity in the evidence heard and read. The reception of the Report has so far as can be judged from early comments in the medical and public press been enthusiastically favourable.

Some of the outstanding observations made by the Committee are worthy of record without comment. Readers may individually agree or disagree with the Committee's conclusions, but they should remember that the Committee heard the evidence, weighed it in an impartial balance and gives its judgment with impartiality, without fear or favour, and most important of all without one "dissenting judgment." Not every Court of Law achieves the same unity of judicial opinion. In brief, here are the conclusions :—

A successful National Health Service depends on Medical Education. Many of the necessary reforms in Medical Education depend upon a change of outlook on the part of teachers and examiners and upon the development of new teaching methods.

Immediate action is needed on three matters :—

1. Building up an adequate supply of teachers.

2. Financial and other assistance to medical schools for provision of staff, accommodation and equipment that will be needed.

3. Provision of suitable post-graduate courses for existing practitioners.

Other recommendations are :—

That every Medical School should be an integral part of a University. This involves radical changes in the relations between the University of London and the London Teaching Hospitals. It also carries with it the cessation of training medical students by the extra-mural schools in Scotland.

In order to remedy defects in the staffing of Clinical Departments it will be necessary

(*a*) To create more whole-time appointments in all grades, senior, intermediate and junior.

(*b*) To adopt new machinery for selecting clinical teachers and members of staffs of teaching hospitals and for diminishing "in-breeding."

(*c*) To pay salaries to clinical teachers for defined duties.

(*d*) To re-organize the educational work of clinical departments so that each such department may have an academic head responsible for the general organization and direction of teaching and research.

As regards whole-time appointments, the general body of evidence is in favour of more whole-time teaching appointments in clinical subjects. It is therefore recommended that every Medical School should have whole-time professors in the departments of general medicine, general surgery and obstetrics and gynæcology. But it is not proposed that clinical teaching should be solely in hands of professors and whole-time teachers. Specialists who are part-time teachers have essential contributions to bring to the training of medical students.

The Report considers that training has, in some instances, suffered because too many students have been attached to an individual teacher for bedside teaching. The number should be limited to six or eight. In every medical teaching centre an adequate staff of non-medical technical assistants is essential. Universities should be responsible for recruiting, training and providing reasonable amenities for them.

The appointment of Regius Professors by the Crown should cease and be transferred to the authorities of the Universities concerned.

*Students.*—Co-education of men and women should be the rule, and unsuitability for a medical career should be the sole barrier to admission to a Medical School.

Financial grants to medical students should be adequate in amount, should cover the whole period of training, and should include cost of maintenance throughout.

The selection of students should not be based on examination results alone. Selection should be based on the candidate having received a good general education, on confidential reports on his previous career and on the impressions gained at an interview.

Moreover, selection machinery must extend to the early weeding-out of students who prove unsuitable.

There are many observations on the number of students at various centres, the upshot of which is the desirability of reducing the number of students admitted to Aberdeen, Edinburgh and Glasgow, and the geographical re-distribution of medical schools in London.

The University of Oxford is advised to develop a small clinical school aiming at producing "teachers, investigators and consultants, rather than general practitioners."

The University of Cambridge is advised that the building up of active post-graduate clinical departments should precede the establishment of an undergraduate clinical school.

*Pre-Clinical Studies.*—It is held that training in physical and biological sciences is best begun at school, but this will involve changes in the school curriculum and examinations, as well as increased and better teaching in these sciences at school. Training in the physical and biological sciences should be continued at the Medical School in close association with the training in anatomy and physiology.

There is urgent need for a new view-point of the part of teachers of pre-clinical subjects and for the drastic elimination from their teaching and the examinations of a mass of detailed information.

*Clinical Studies.*—Since the majority of students become general practitioners, clinical teaching must be largely directed to the problems that most frequently face them in general practice.

General medicine, minor ailments, common diseases, early stages of disease, chronic diseases, infectious diseases and rehabilitation must receive more attention.

The surgical part of the undergraduate course should be closely correlated with other undergraduate work.

The technical details of operative measures are matters for post-graduate study. Undergraduate training should be directed to the recognition, early treatment, personal, social and economic effects of those surgical conditions commonly encountered in general practice (acute injuries, acute infections, abdominal emergencies and malignant disease).

Social and preventive medicine, child health and disease, psychological medicine and maternity work must receive more attention.

After passing the Final Qualifying examination every student should be required to serve as a junior house officer for a period of twelve months in one or more approved hospitals before admission to the Medical Register and being permitted to enter independent medical practice.

*Final Examinations.*—In order that the burden of final examinations may be relieved and students encouraged to pay proper attention to all parts of the training "internal" departmental examinations in suitable subjects should be instituted as a complement to the final examination.

One effect of the recommendations, if adopted, will be the abolition of the qualifying examination as at present conducted by Licensing Corporations which have no responsibility for teaching.

---

This review does not deal with Part II of the Report "Postgraduate Medical Education and Research."

